# Towards Sustainable Aquafeeds: Complete Substitution of Fish Oil with Marine Microalga *Schizochytrium* sp. Improves Growth and Fatty Acid Deposition in Juvenile Nile Tilapia (*Oreochromis niloticus*)

**DOI:** 10.1371/journal.pone.0156684

**Published:** 2016-06-03

**Authors:** Pallab K. Sarker, Anne R. Kapuscinski, Alison J. Lanois, Erin D. Livesey, Katie P. Bernhard, Mariah L. Coley

**Affiliations:** Environmental Studies Program, Dartmouth College, Hanover, NH 03755, United States of America; Universidade de Vigo, SPAIN

## Abstract

We conducted a 84-day nutritional feeding experiment with dried whole cells of DHA-rich marine microalga *Schizochytrium* sp. (Sc) to determine the optimum level of fish-oil substitution (partial or complete) for maximum growth of Nile tilapia. When we fully replaced fish oil with *Schizochytrium* (Sc100 diet), we found significantly higher weight gain and protein efficiency ratio (PER), and lower (improved) feed conversion ratio (FCR) and feed intake compared to a control diet containing fish oil (Sc0); and no significant change in SGR and survival rate among all diets. The Sc100 diet had the highest contents of 22:6n3 DHA, led to the highest DHA content in fillets, and consequently led to the highest DHA:EPA ratios in tilapia fillets. *Schizochytrium* sp. is a high quality candidate for complete substitution of fish oil in juvenile Nile tilapia feeds, providing an innovative means to formulate and optimize the composition of tilapia juvenile feed while simultaneously raising feed efficiency of tilapia aquaculture and to further develop environmentally and socially sustainable aquafeeds. Results show that replacing fish oil with DHA-rich marine Sc improves the deposition of n3 LC PUFA levels in tilapia fillet. These results support further studies to lower *Schizochytrium* production costs and to combine different marine microalgae to replace fish oil and fishmeal into aquafeeds.

## Introduction

Aquaculture has been rapidly expanding all over the world in the last few decades, at an average rate of 8–10% per year [1]. Between 1980 and 2010, aquaculture contribution to global fishery output for human consumption rose from nine percent to 47 percent and its use of artificial feeds rose from 50 to 66 percent of production [2,3]. Analysts expect aquaculture to provide an additional 23 million tonnes of aquatic foods by 2030 to maintain the current per-capita level of consumption of aquatic foods for a growing global population [3]. Responsible expansion of aquafeeds, *inter alia*, requires finding sustainable alternatives to fishmeal and fish oil ingredients, of which aquaculture is the largest user. Aquaculture feeds currently use over 80% of the world’s fishmeal and fish oil [3], which are extracted from small ocean-caught fish. To date, fish oils (FO) are still regarded as good lipid sources of aquafeed formulations [4]. However, due to the short-fall from wild-harvested marine fish stocks for the production of fish oil [3], alternative lipid sources for the production of fish feed are essential to meet the demand of the growing aquaculture industry [5].

This research project focuses on reformulation of diets for Nile tilapia (*Oreochromis niloticus*), the second most farmed fish worldwide [6] and the most widely farmed in the United States [7]. This species is pre-adapted to thrive on fish-ingredient-free aquafeed because of its low trophic status. Commercial diets for tilapia have already greatly reduced inclusion levels of fish oil by replacing them with terrestrial vegetable oils, which have relatively low prices and large production volumes. Scientists have reported considerable success in partially or totally replacing fish oil with vegetable oil in many fish species [8, 9]. Several studies have shown that replacement of high-quality fish oil with vegetable oil sources negatively affects growth performance, fatty acid profile or health parameters of fish, such as gilthead seabream (*Sparus aurata*) [10] and European sea bass (*Dicentrarchus labrax* L.) [11], the magnitude of which depends on the source and level of replacement. Moreover, unbalanced essential amino acids, low levels of n3 PUFAs, lack of DHA and EPA, and high levels of anti-nutritional factors [12] limit the inclusion rates of terrestrial plant ingredients, even in diets for omnivorous species like tilapia [13,14].

Recent commercial-scale production of microalgae for biofuels and human nutritional supplements has also stimulated interest in microalgae for animal feeds [15]-[16]. Increasing attention has focused on marine microalgae for aquaculture feeds because of their elevated fatty acid profiles. Additionally, microalgae are at the base of the aquatic food chain that produce the food resources that tilapia are adapted to consume. In contrast to terrestrial plant sources, microalgae are relatively high in essential long chain n-3 polyunsaturated fatty acids (n3 LC PUFA) such as DHA (C22:6n3) and EPA (C20:5n3), which are important both for maintaining fish health and imparting neurological, cardiovascular and anticancer benefits to humans [17,18]. Our recent evaluation of a marine microalga, *Schizochytrium* sp. (Sc), in tilapia found significantly higher digestibility of lipid and all unsaturated fatty acid fractions from Sc compared to freshwater microalgae [19]. This was the first study to report that dried whole cells of Sc are a highly digestible lipid source for Nile tilapia. However, the response of fish growth performance, feed utilization efficiency, and fatty acid deposition in muscle to dietary inclusion of Sc is unknown. Building on our previous findings on digestibility of Sc in tilapia [19], the study reported below determined whether whole-cell Sc is a quality fish oil substitute in Nile tilapia feed. We conducted a nutritional feeding trial with diets containing dried whole cells of this microalga designed to identify the optimum level of fish oil substitution (partial or complete) to support high growth and feed efficiency, and to measure whether inclusion of dried whole-cell Sc can raise n3 LC PUFAs deposition in juvenile tilapia fillets.

## Materials and Methods

### Experimental design, fish rearing and feeding

We obtained Nile tilapia (*O*. *niloticus*) juveniles from Americulture Inc. (Animas, New Mexico, 88020, USA). We conducted this experiment in a temperature controlled wet lab at the Life Sciences Center, Dartmouth College (Hanover, NH, USA) using fifteen indoor, static-water 114-L cylindro-conical tanks. We filled each tank with charcoal filtered de-chlorinated tap water and provided aeration through an air stone diffuser via a low-pressure electrical blower. Each tank contained bio-ball and sponge biological filters. Prior to the start of the experiment, we randomly assigned 40 tilapia to each tank, with an initial mean weight of 1.52 ± 0.2 g/fish and accustomed the fish to a photoperiod cycle of 10 h light and 14 h dark. We acclimated fish to the experimental conditions for two weeks before starting the experiment, during which we fed them the control diet (see section, feed formulation and pellet preparation). We randomly allocated the five experimental diets to 15 tanks and fed each diet to three replicate tanks (n = 3) in a completely randomized design. We hand-fed fish three times daily at 0930, 1300 and 1700h for 84 days. At the start of the trial, we administered feed at a rate of 10% of body weight. We recorded the daily satiation ration and used this to guide a gradual reduction of the feeding rate to 4% of body weight at the end of the trial, following the approach of Hussein et al. 2012 and Karapanagiotidis et al. 2007 for tilapia [20,21], and NRC 2011 [4]. The experimental design and fish use protocol were approved by the Institutional Animal Care and Use Committee (IACUC) of Dartmouth College.

All water quality parameters, monitored during the course of the study, confirmed that we maintained excellent conditions for tilapia. We exchanged 10–15% of the tank water each week. We kept water temperature throughout the experiment within the range 26.4–28.2˚C with a thermostat-regulated, immersion heater in each tank (Hagen Marina Submersible Pre-Set Aquarium Heater, 150W, model UPC 015561112345). The range of values for other variables were pH 7.17 to 7.60, dissolved oxygen 6.18 to 7.13 mg L^-^1, nitrite 0.10 to 0.20 mg L^-^1, and total ammonia nitrogen 0.23 to 0.53 mg L^-^1.

### Feed formulation and pellet preparation

We formulated five iso-nitrogenous (38% crude protein), iso-energetic (14 kJ/g) and iso-lipidic (10% lipid) experimental diets, following the requirements for optimum growth of juvenile Nile tilapia [4]. We prepared a control diet for juvenile tilapia, adapted from a proven high quality formulation [22]. The diets differed from each other in their relative amounts of fish oil (menhaden-derived) and dried whole cells of *Schizochytrium* sp. (Sc). We used wheat flour (only 11% protein) as filler [20] and all experimental diets were iso-nitrogenous, iso-energetic, iso-lipidic composition, and also balance protein/lipid ratio differences between fish oil and its replacer (Sc) in the diets. We designated control feed containing fish oil (Sc0), and substituted 25% fish oil (Sc25), 50% fish oil (Sc50), 75% fish oil (Sc75), and 100% fish oil (Sc100) with dried whole cells of *Schizochytrium* sp. (Table 1). We obtained dried *Schizochytrium* sp. from Algamac™, Aquafauna Bio-marine, Inc., Hawthorne, CA, USA. We obtained menhaden fish oil from Double Liquid Feed Service, Inc., Danville, IL, USA. We produced the diets in accordance with our previous work [19], by weighing and mixing oil and dry ingredients in a stand mixer (Hobart Corporation, Tory, OH, USA) for 15 min; then blending water (330 ml kg^-1^ diet) into the mixture to attain a texture appropriate for pelleting; and running each diet through a meat grinder (Panasonic, MK-G20NR) to create 2 mm-diameter pellets. After pelleting, we dried the diets to a moisture content of 80–100 g kg^-1^ under a chemical fume hood at room temperature for 12 h and then stored the finished diets at -20°C. Table 2 reports the proximate composition, gross energy, and amino acid profiles of dried Sc and Table 3 reports the fatty acid profile of dried Sc and menhaden fish oil. Table 4 reports the fatty acid profiles of the five experimental diets.

**Table 1:** Formulation (g/100g diet) and proximate composition (%) and essential amino acids (% in the weight of diet as is) of five experimental diets for juvenile tilapia.

			Diet		
Ingredient	Control (Sc0)	Sc25	Sc50	Sc75	Sc100
Fish meal*	20	20	20	20	20
Corn gluten meal	20	20	20	20	20
Soybean meal	20	20	20	20	20
Wheat flour	26.25	24.5	22.75	20.5	19.15
CaH_2_PO_4_	0.75	0.75	0.75	0.75	0.75
Vitamin mix^1^	1	1	1	1	1
Mineral mix^2^	1	1	1	1	1
Fish oil	9	6.75	4.5	2.25	0
*Schizochytrium* sp.	0	4	8	12.5	16.1
Choline chloride	2	2	2	2	2
Proximate composition (%)					
Dry matter	88.2	89	90.2	89.4	91.2
Crude protein	38.2	38.6	39.3	39.1	39.3
Lipid	11.1	11.1	10.6	10.7	10.2
Ash	7	7.4	7.2	8	8.6
NFE	31	31.8	32.2	31.4	32
Gross energy (kJ g-1)	14	14.1	14.2	14	14.1
Amino acids (% in the weight of diet as is)					
Arginine	2	2	2.1	2	2.2
Lysine	2	2	2.1	1.8	2.1
Isoleucine	1.4	1.4	1.6	1.4	1.5
Leucine	3.7	3.9	4.1	3.7	4.2
Histidine	0.8	0.8	0.8	0.8	0.8
Methionine	0.8	0.8	0.9	0.9	0.8
Cystine	0.5	0.5	0.5	0.5	0.5
Phenyle alanine	1.8	1.9	2	1.8	2.1
Threonine	1.3	1.3	1.5	1.2	1.5
Tryptophan	0.2	0.2	0.2	0.1	0.1
Valine	1.6	1.6	1.8	1.6	1.8

^1^Vitamin premix (mg kg^-^1 dry diet unless otherwise stated):vitamin A (as acetate), 7500 IU kg^-^1 dry diet; vitamin D3 (as cholecalcipherol), 6000 IU kg^-^1 dry diet; vitamin E (as DL-a-tocopherylacetate), 150 IU kg^-^1 dry diet; vitamin K (as menadione Na-bisulphate), 3; vitamin B12 (as cyanocobalamin), 0.06; ascorbic acid (as ascorbyl polyphosphate), 150; D-biotin, 42; choline (as chloride), 3000; folic acid, 3; niacin (as nicotinic acid), 30; pantothenic acid, 60; pyridoxine, 15; riboflavin, 18; thiamin, 3

^2^Mineral premix (mg kg^-^1 dry diet unless otherwise stated):ferrous sulphate, 0.13; NaCl, 6.15; copper sulphate, 0.06; manganese sulphate, 0.18; potassium iodide, 0.02; zinc sulphate, 0.3; carrier (wheat middling or starch).

*Omega Protein, Inc. Houston, Texas 77042, as manufacturer specification, the guaranteed gross composition analysis: crude protein, 60%; crude fat, 6%; fiber, 2%.

**Table 2:** Proximate chemical composition, gross energy, essential, and non-essential amino acids of whole dried cell of *Schizochytrium* sp.

	Ingredient
Composition	*Schizochytrium* sp.
Dry matter	96.5
Crude protein	11.9
Lipid	54.1
Ash	8.7
Crude fibre	2.4
Energy	17.7
Essential amino acids (% in the weight of ingredient as is)	
Arginine	0.8
Lysine	0.5
Isoleucine	0.4
Leucine	0.7
Histidine	0.3
Methionine	1.2
Phenylealanine	0.4
Threonine	0.4
Tryptophan	0.2
Valine	0.6
Non-essential amino acids ((% in the weight of ingredient as is)	
Aspartic acid	1.2
Serine	0.4
Glutamic acid	1.9
Glycine	0.5
Tyrosine	0.3
Alanine	0.8
Hydroxyproline	0.0
Proline	0.5

**Table 3:** Fatty acid content (% of total fatty acids) of lipid sources (menhaden fish oil and whole cell dried *Schizochytrium* sp) used in the experimental diets.

	Lipid source	
Fatty acids (% of TFA)	Fish oil	*Schizochytrium* sp
14:00	8.1	9.3
15:00	0.6	0.5
16:00	17.9	24.4
17:00	0.6	ND
18:00	3.1	0.5
20:00	0.2	0.1
22:00	0.1	0.1
24:00	ND	ND
Total SFA	6.6	9.2
16:1n9	0.2	ND
16:1n7	13.9	0.2
18:1n9	5.2	0.1
18:1n7	3.3	ND
20:1n9	0.6	ND
20:1n7	0.2	ND
22:1n11	ND	ND
22:1n9	0.1	ND
24:1n9	0.4	1.4
Total MUFA	23.9	1.7
18:2n6	1.5	ND
18:3n6	0.3	0.2
20:2n6	0.2	ND
20:3n6	0.2	0.3
20:4n6 ARA	1.3	1.4
22:4n6	0.3	0.1
22:5n6	0.5	15.8
Total n6 PUFA	4.3	17.8
18:3n3 ALA	1.5	ND
18:4n3	2.7	0.6
20:3n3	0.2	0.1
20:4n3	1.4	0.8
20:5n3 EPA	14.9	0.8
22:5n3	2.6	0.4
22:6n3 DHA	13	43.2
Total n3 PUFA	36.3	45.9
Total PUFA	40.6	63.7
Total n6 LCPUFA	2.5	17.6
Total n3 LCPUFA	32.1	45.3
n3:n6 PUFA ratio	8.4	2.6

SFA, saturated fatty acids (sum of all fatty acids without double bonds); MUFA, monounsaturated fatty acids (sum of all fatty acids with a single bond); PUFA, polyunsaturated fatty acids (sum of all fatty acids with ≥2 double bonds); n6 PUFA, omega 6 polyunsaturated fatty acids (18:2, 18:3, 20:2, 20:3, 20:4, 22:4, 22:5); n6 LCPUFA, omega 6 long chain polyunsaturated fatty acids (20:2, 20:3, 20:4, 22:4, 22:5), n3 PUFA, omega 3 polyunsaturated fatty acids (18:3, 18:4, 20:3, 20:4, 20:5, 22:5, 22:6); n3 LCPUFA, omega 3 long chain polyunsaturated fatty acids (20:3, 20:4, 20:5, 22:5, 22:6); EPA, eicosapentaenoic acid; DHA, docosahexaenoic acid; ND, not detectable (<0.1% of total fatty acids).

**Table 4:** Fatty acid content (% of total fatty acids) of experimental diets.

			Diet		
Fatty acids (% of TFA)	Control (Sc0)	Sc25	Sc50	Sc75	Sc100
14:00	7.6	8	8.2	8	8
15:00	0.5	0.6	0.5	0.5	0.5
16:00	18.4	19.6	20.7	21.7	22.8
17:00	0.6	0.5	0.4	0.3	0.1
18:00	3.2	3.0	2.4	1.8	1.3
20:00	0.2	0.2	0.2	0.1	0.2
22:00	0.2	0.1	0.1	0.1	0.2
24:00	0.1	0.1	0.1	0.1	0.2
Total SFA	30.8	32.1	32.6	32.6	33.3
16:1n9	0.2	0.2	0.2	ND	ND
16:1n7	11	9.1	6.8	4.1	2.0
18:1n9	7.8	6.7	5.6	5.0	4.2
18:1n7	2.9	2.4	1.8	1.2	0.7
20:1n9	0.5	0.4	0.3	0.2	0.1
20:1n7	0.2	0.1	0.1	0.3	ND
22:1n11	ND	ND	ND	ND	ND
22:1n9	ND	ND	ND	ND	ND
24:1n9	0.3	0.5	0.6	0.9	1.0
Total MUFA	23	19.4	15.4	11.7	8.0
18:2n6	10.1	9.4	9.1	9.2	9.6
18:3n6	0.3	0.2	0.2	0.2	0.1
20:2n6	0.2	0.1	0.1	ND	ND
20:3n6	0.2	0.2	0.2	ND	0.3
20:4n6 ARA	1.2	1.3	1.2	1.2	1.3
22:4n6	0.2	0.2	0.1	0.1	0.1
22:5n6	0.8	3	5.7	8.5	10.8
Total n6 PUFA	13	14.4	16.6	19.2	22.2
18:3n3 ALA	1.6	1.4	1.1	0.9	0.7
18:4n3	1.9	1.6	1.2	0.8	0.4
20:3n3	0.2	0.1	0.1	0.1	ND
20:4n3	1.0	1.0	0.8	0.8	0.6
20:5n3 EPA	11.1	9.22	7.0	4.6	2.5
22:5n3 DPA	2.1	1.8	1.3	1.0	0.6
22:6n3 DHA	10.4	15	20.7	26.2	30.8
Total n3 PUFA	28.3	30.12	32.2	34.4	35.6
Total PUFA	41.3	44.52	48.8	53.6	57.8
Total n6 LCPUFA	2.6	4.8	7.3	9.8	12.5
Total n3 LCPUFA	24.8	27.12	29.9	32.7	34.5
n3:n6 PUFA ratio	2.2	2.1	1.9	1.8	1.6

SFA, saturated fatty acids (sum of all fatty acids without double bonds); MUFA, monounsaturated fatty acids (sum of all fatty acids with a single bond); PUFA, polyunsaturated fatty acids (sum of all fatty acids with ≥2 double bonds); n6 PUFA, omega 6 polyunsaturated fatty acids (18:2, 18:3, 20:2, 20:3, 20:4, 22:4, 22:5); n6 LCPUFA, omega 6 long chain polyunsaturated fatty acids (20:2, 20:3, 20:4, 22:4, 22:5), n3 PUFA, omega 3 polyunsaturated fatty acids (18:3, 18:4, 20:3, 20:4, 20:5, 22:5, 22:6); n3 LCPUFA, omega 3 long chain polyunsaturated fatty acids (20:3, 20:4, 20:5, 22:5, 22:6); EPA, eicosapentaenoic acid; DHA, docosahexaenoic acid; ND, not detectable (<1% of total fatty acids).

### Biological sampling procedures, fillet preparations and growth measurements

We bulk weighed the fish at the beginning of the experiment, and then every 3 weeks until the end of the experiment (84 days). We stopped feeding the fish for 24 h prior to each bulk weight-sampling event. We sampled 5 fish per tank at day 42 (middle) and day 84 (final) for fillet fatty acid composition analysis. We immediately euthanized the fish by single cranial pithing, filleted the fish from a standardized dorso-anterior landmark, packaged in sterile polythene bags (Whirl-pak, Naso, Fort Atkinson, Wisconsin) and stored frozen (-20°C) until fatty acid analysis. At the end of the feeding experiment, we sampled 10 fish per tank, pooled, ground into a homogeneous slurry, freeze-dried, reground and stored at—20°C until analyzed for the whole-body proximate analysis.

We determined the dietary effects on growth by evaluating final weight, weight gain, feed conversion ratio (FCR), specific growth rate (SGR), protein efficiency ratio (PER) and percent survival. We calculated the indices as follows: Weight gain (g) = final weight–initial weight; FCR, feed conversion ratio = feed intake (as fed basis)/ weight gain; SGR, specific growth rate (%/day) = 100 x (ln final wet weight (g)–ln initial wet weight (g)) / Time (days); PER, protein efficiency ratio = weight gain (g)/protein fed (g); and % survival rate = (Final number of fish / Initial number of fish) x 100.

### Biochemical analysis

We sent the five types of samples (pure microalgae, fish oil, diets, wholebody and fillet) to New Jersey Feed Laboratory, Inc. (Ewing, NJ, USA) for the following types of analysis [23]: moisture (AOAC, 930.15), crude protein (AOAC 990.03), lipid (AOAC 920.39), ash (AOAC 942.05), crude fiber (AOAC 1978.10), The nitrogen-free extract (NFE; i.e. carbohydrate) was determined by difference: NFE = 100 − (% protein + % lipid + % fiber + %moisture + % ash), energy (automated oxygen bomb calorimeter), amino acids (high-performance liquid chromatography, HPLC analysis, via AOAC methods 994.12, 985.28, 988.15, and 994.12) and fatty acid (fatty acids methyl esters, FAME analysis, via AOAC method 963.22.). Table 3 reports the fatty acid content of the two lipid sources used, menhaden fish oil and Sc whole dried cells; and Table 4 reports the fatty acid content of the five experimental diets.

### Statistical analysis

We conducted one-way analysis of variance (ANOVA) of growth performance and feed utilization parameters, whole body proximate composition, fillet fatty acid deposition and, when significant differences were found, compared the treatment means using Tukey’s test of multiple comparisons with 95% level of significance. Data were expressed as the mean ± SE of three replicates. We conducted a repeated measure analysis within the general linear model (GLM) framework for 42 and 84 days of fillet fatty acids (%TFA) data to determine whether there were differences among dietary treatments, sampling time, or main effect interactions (diet x time). We carried out all statistical analyses using the IBM Statistical Package for the Social Sciences (SPSS) program for Windows (v. 21.0, Armonk, NY, USA). We computed the Pearson correlation coefficient between Sc inclusion level in a diet and tissue deposition of DHA, weight gain and FCR data.

## Results

We conducted a 84-day growth experiment with dried whole-cells of Sc and found (Table 5): significantly higher weight gain (g) and protein efficiency ratio (PER), and lower (improved) feed conversion ratio (FCR) and feed intake when fish oil was fully replaced by Sc (Sc100 diet) compared to control diet containing fish oil (Sc0 diet). Tilapia appeared healthy at the end of the experiment, and showed no difference in SGR and survival rate among all diets. Weight gain was in the range of 23.8–27.3 g. Feed intake ranged from 23.6 to 27.0 g/fish. Feed conversion ratios (FCR) were within the range 0.9–1.1 and protein efficiency ratios (PER) were within the range 2.4–3.1 among all dietary treatments. We found a strong proportional linear relationship between the Sc content of the diet and weight gain (y = 0.0193x + 23.895; r = 0.970; P< 0.01). The FCR decreased (improved) as the dietary Sc content increased, showing an inverse relationship (y = -0.013x + 1.101; r = 0.970; P<0.05).

**Table 5:** Initial weight, final weight, weight gain, percentage weight gain, feed conversion ratio (FCR), specific growth rate (SGR), protein efficiency ratio (PER), feed intake, and survival rate of tilapia fed experimental diets.

		Diet^1^				ANOVA	
	Sc0	Sc25	Sc50	Sc75	Sc100	*F* value	*P* value
Initial weight (g)	1.4 ± 0.1	1.42 ± 0.1	1.7 ± 0.2	1.5 ± 0.1	1.6 ± 0.1	1.5	0.27
Final weight (g)	25.3 ± 0.3bc	26.4 ± 0.4bc	27.2 ± 0.7ab	27.4 ± 0.3ab	28.8 ± 0.2a	9.9	<0.01
Weight gain (g)^2^	23.8 ± 0.4bc	24.9 ± 0.3bc	25.5 ± 0.7ab	25.8 ± 0.2ab	27.3 ± 0.2a	9.3	<0.01
FCR^3^	1.1 ± 0.0bc	1.0 ± 0.0bc	1.0 ± 0.1ab	0.9 ± 0.0ab	0.9 ± 0.0a	10.2	<0.01
SGR^4^	3.4 ± 0.1	3.5 ± 0.1	3.3 ± 0.0	3.4 ± 0.1	3.5 ± 0.1	0.8	0.52
PER^5^	2.4 ± 0.1bc	2.6 ± 0.1bc	2.7 ± 0.2ab	2.8 ± 0.1ab	3.1 ± 0.1a	9.6	<0.01
Feed intake (g/fish)	27.0 ± 0.3a	25.9 ± 0.4ab	25.1 ± 0.6bc	24.9 ± 0.2bc	23.6 ± 0.2c	10.4	<0.01
%Survival rate^6^	92.7 ± 0.37	95.2 ± 1.5	95.8 ± 0.8	92.3 ± 0.2	96.3 ± 2.6	0.9	0.57

Values are means of ±SE of three replicate groups (n = 3)

^1^Mean values not sharing a superscript letter in the same row differ significantly (P < 0.05).

^2^Weight gain (g) = final wet weight–initial wet weight

^3^FCR, feed conversion ratio = feed intake/ weight gain

^4^Specific growth rate SGR (%/day) = 100 x (ln final wet weight (g)–ln initial wet weight (g)) / Time (days);

^5^PER, protein efficiency ratio = weight gain (g)/protein fed (g)

^6^%Survival = (Final number of fish / Initial number of fish) x 100.

The whole body proximate composition of Nile tilapia fillets did not differ among dietary treatments (data not shown). This included moisture (ranged from 70.5 to 71.0%), crude protein (ranged from 16.1 to 16.6.0%), ash (ranged from 4.5 to 4.8%) and total lipid (ranged from 6.2 to 6.7%).

The fillet fatty acids composition (% of total fatty acids) was significantly influenced either by the dietary treatment or the length of the experiment or both factors (Table 6). With the exception of 18:0, all SFA fractions showed significant time effects and were higher at the middle (42 days) than at the end of the experiment (84 days). The composition of four SFA fractions (15:0, 18:0, 20:0, and 22:0) did not differ across dietary treatments. One SFA, palmitic acid (16:0), had the highest final concentration in the fillet irrespective of dietary treatment, as well as significantly higher amounts deposited in the flesh of tilapia fed the Sc100 diet compared to fish fed the Sc0 diet (P<0.01). It also showed a significant diet and time interaction (P = 0.03). Concentrations of 14:0 and total saturated fatty acid (SFA) were significantly higher in the Sc100-fed fish than in Sc0-fed fish at 42 days, as well as at 84 days (P<0.01). These results were probably due to the higher supply of these two components in the Sc100 diet compared to the Sc0 diet (Table 4).

**Table 6:** Fatty acid (% of total fatty acids) content of fillets from Nile tilapia fed experimental diets for 84 days and 42 days sampling events; average ± SE for 15 fish per diet (5 fish/replicate with 3 replicates/diet)^§^.

	Control (Sc0)		Sc25		Sc50		Sc75		Sc100		*P* value‡		
Fatty acid (% TFA)	84 days	42 days	84 days	42 days	84 days	42 days	84 days	42 days	84 days	42 days	Diet	Time	Diet x Time interaction
14:00	5.6 ± 0.1^b^	7.6 ± 0.7^b^	6.1 ± 0.9^b^	8.8 ± 0.2^ab^	6.1 ± 0.3^ab^	9.6 ± 0.4^a^	6.9 ± 0.6^b^	10.5 ± 0.2^a^	7.5 ± 0.3^b^	9.6 ± 0.1^a^	0.01	<0.01	0.12
15:00	0.6 ± 0.0	0.7 ± 0.0	0.5 ± 0.0	0.8 ± 0.0	0.5 ± 0.0	0.8 ± 0.0	0.5 ± 0.0	0.6 ± 0.0	0.4 ± 0.0	0.6 ± 0.0	0.06	<0.01	0.47
16:00	19.9 ± 0.1^c^	28.6 ± 1.1^b^	24 ± 2.0^bc^	32.2 ± 0.9^b^	24.0 ± 0.6^bc^	38.8 ± 0.4^a^	25.6 ± 2.4^bc^	41.2 ± 0.5^a^	30.0 ± 1.6^b^	42.3 ± 0.6^a^	<0.01	<0.01	0.03
17:00	0.7 ± 0.0^a^	0.8 ± 0.0^a^	0.6 ± 0.0^b^	0.8 ± 0.0^a^	0.5 ± 0.0^ab^	0.7 ± 0.0^a^	0.4 ± 0.0^c^	0.5 ± 0.0^bc^	0.3 ± 0.0^c^	0.4 ± 0.0^c^	<0.01	<0.01	0.21
18:00	6.9 ± 0.2	7.9 ± 0.2	6.1 ± 0.5	8.0 ± 0.5	5.7 ± 0.2	8.3 ± 0.3	5.5 ± 0.4	8.2 ± 0.2	6.1 ± 0.3	7.9 ± 0.3	0.99	<0.01	0.65
20:00	0.3 ± 0.0	0.3 ± 0.0	0.3 ± 0.0	0.4 ± 0.0	0.3 ± 0.0	0.3 ± 0.0	0.2 ± 0.0	0.3 ± 0.0	0.3 ± 0.0	0.4 ± 0.0	0.41	0.08	0.2
22:00	0.1 ± 0.0	0.1 ± 0.0	0.1 ± 0.0	0.1 ± 0.0	0.1 ± 0.0	0.1 ± 0.0	ND	0.1 ± 0.0	0.1 ± 0.0	ND			
24:00:00	0.1 ± 0.0	ND	0.1 ± 0.0	0.1 ± 0.0	0.1 ± 0.0	ND	0.1 ± 0.0	0.1 ± 0.0	0.1 ± 0.0	0.1 ± 0.0	0.99	<0.01	0.65
Total SFA	34.2 ± 0.2^c^	46.0 ± 2.3^b^	37.8 ± 3.6^c^	51.2 ± 1.8^b^	37.3 ± 0.6^c^	58.6 ± 0.6^a^	39.2 ± 3.5^c^	61.5 ± 0.5^a^	44.8 ± 2.3^b^	61.3 ± 0.9^a^	<0.01	<0.01	0.07
16:1n9	0.4 ± 0.2^a^	0.4 ± 0.0	0.3 ± 0.0^ab^	0.4 ± 0.0	0.3 ± 0.0^ab^	0.3 ± 0.0	0.3 ± 0.0^ab^	0.3 ± 0.0	0.2 ± 0.0^b^	0.2 ± 0.1	0.13	0.72	0.96
16:1n7	8.7 ± 0.1^b^	11.1 ± 0.3^a^	6.7 ± 1.1^c^	9.5 ± 0.1^a^	5.8 ± 0.2^c^	7.6 ± 0.3^b^	5.3 ± 0.6^c^	5.8 ± 0.3^c^	2.9 ± 0.0^d^	2.9 ± 0.2^d^	<0.01	<0.01	0.09
18:1n9	9.8 ± 0.2^b^	14.4 ± 0.0^a^	9.9 ± 0.6^b^	12.8 ± 0.4^a^	8.3 ± 0.5^b^	12.1 ± 0.6^a^	9.8 ± 1.0^b^	12.2 ± 0.6^a^	8.3 ± 0.4^b^	9.1 ± 0.6^b^	0.01	<0.01	0.05
18:1n7	4.1 ± 0.1^b^	5.1 ± 0.0^a^	3.5 ± 0.3^bc^	4.5 ± 0.1^a^	3.1 ± 0.1^bc^	4.0 ± 0.1^b^	2.8 ± 0.2^c^	3.3 ± 0.1^bc^	2.1 ± 0.0^c^	2.5 ± 0.0 ^c^	<0.01	<0.01	0.15
20:1n9	0.6 ± 0.0^b^	0.8 ± 0.0^a^	ND	0.8 ± 0.0^a^	0.5 ± 0.0^b^	0.6 ± 0.0^b^	0.5 ± 0.0^b^	0.7 ± 0.0^a^	0.4 ± 0.0^b^	0.4 ± 0.0^b^	<0.01	<0.01	0.08
20:1n7	0.2 ± 0.0^a^	0.1 ± 0.0^b^	0.2 ± 0.0^a^	0.1 ± 0.0^b^	0.1 ± 0.0^b^	ND	0.1 ± 0.0^b^	0.1 ± 0.0^b^	ND	ND	<0.01	<0.01	<0.01
24:1n9	0.3 ± 0.0^a^	0.1 ± 0.0^b^	0.2 ± 0.0^ab^	0.1 ± 0.0^b^	0.1 ± 0.0^b^	ND	0.1 ± 0.0^b^	0.1 ± 0.0^b^	ND	ND	0.03	<0.01	<0.01
Total MUFA	24.1 ± 0.3^b^	32.0 ± 0.7^a^	21.2 ± 2.2^b^	28.2 ± 0.4^a^	18.2 ± 0.7^bc^	24.6 ± 0.9^b^	18.9 ± 1.9^bc^	22.5 ± 1.1^b^	14.0 ± 0.6^c^	15.1 ± 0.9^c^	<0.01	<0.01	0.13
18:2n6	8.6 ± 0.0^a^	5.0 ± 0.8^b^	6.9 ± 0.7^b^	4.2 ± 0.4^bc^	7.2 ± 0.2^a^	2.8 ± 0.2^c^	6.6 ± 0.2^b^	2.3 ± 0.1^c^	5.8 ± 0.3^b^	4.0 ± 0.6^c^	<0.01	<0.01	0.18
18:3n6	0.3 ± 0.0^a^	ND	0.2 ± 0.0^ab^	0.1 ± 0.0^b^	0.2 ± 0.0^ab^	ND	0.2 ± 0.0^ab^	ND	0.1 ± 0.0^b^	ND	0.02	<0.01	0.03
20:2n6	0.4 ± 0.0^a^	0.2 ± 0.0 ^ab^	0.3 ± 0.0^a^	0.2 ± 0.0^ab^	0.3 ± 0.0^a^	ND	0.3 ± 0.0 ^a^	0.1 ± 0.0^b^	0.2 ± 0.0^ab^	0.1 ± 0.0^b^	<0.01	<0.01	<0.01
20:3n6	0.4 ± 0.0^a^	0.2 ± 0.0^b^	0.4 ± 0.0^a^	0.2 ± 0.0^b^	0.3 ± 0.0^ab^	ND	0.4 ± 0.0^a^	0.1 ± 0.0^b^	0.3 ± 0.0^ab^	ND	<0.01	<0.01	0.13
20:4n6 ARA	1.7 ± 0.0	0.9 ± 0.0	1.6 ± 0.1	0.9 ± 0.0	1.7 ± 0.1	0.8 ± 0.1	1.5 ± 0.1	0.8 ± 0.0	1.6 ± 0.0	1.1 ± 0.0	0.6	<0.01	0.07
22:4n6	0.5 ± 0.0^a^	0.2 ± 0.0^b^	0.4 ± 0.0^a^	ND	0.4 ± 0.0^a^	ND	0.4 ± 0.0 ^a^	ND	0.3 ± 0.0 ^ab^	ND	0.04	<0.01	0.22
22:5n6	0.8 ± 0.0^d^	0.4 ± 0.0^d^	4.2 ± 1.0^b^	1.5 ± 0.1^c^	5.6 ± 0.2^ab^	2.0 ± 0.2^c^	6.2 ± 1.0^a^	2.3 ± 0.2^c^	7.8 ± 0.5^a^	3.9 ± 0.1^ab^	<0.01	<0.01	<0.01
Total n6 PUFA	12.6 ± 0.1	6.9 ± 1.0	13.9 ± 1.8	7.1 ± 0.5	15.6 ± 0.5	5.6 ± 0.4	15.5 ± 1.4	5.6 ± 0.5	16.1 ± 0.9	9.1 ± 0.6	0.43	<0.01	<0.01
18:3n3 ALA	1.0 ± 0.0 ^a^	0.4 ± 0.0^b^	0.6 ± 0.1^ab^	0.3 ± 0.0^b^	0.7 ± 0.0^ab^	0.1 ± 0.0^c^	0.5 ± 0.0^b^	0.1 ± 0.0^c^	0.3 ± 0.0^b^	0.2 ± 0.1^c^	<0.01	<0.01	<0.01
18:4n3	0.7 ± 0.0^a^	0.4 ± 0.0^b^	0.5 ± 0.0^a^	0.2 ± 0.0^bc^	0.5 ± 0.0^a^	0.1 ± 0.0^c^	0.3 ± 0.0^bc^	0.1 ± 0.0^c^	0.1 ± 0.0^c^	ND	0.03	<0.01	0.1
20:3n3	0.2 ± 0.0^ab^	ND	0.1 ± 0.0^b^	ND	0.2 ± 0.0^ab^	ND	0.1 ± 0.0^b^	ND	0.1 ± 0.0^b^	ND	0.02	<0.01	0.02
20:4n3	0.9 ± 0.0^a^	0.3 ± 0.0^c^	0.6 ± 0.0^b^	0.2 ± 0.0^c^	0.6 ± 0.0^b^	ND	0.5 ± 0.0^a^	0.1 ± 0.0 ^c^	0.3 ± 0.0^c^	ND	<0.01	<0.01	0.03
20:5n3 EPA	3.9 ± 0.1^a^	1.8 ± 0.3^b^	2.2 ± 0.3^b^	1.3 ± 0.1^c^	1.9 ± 0.1^b^	0.7 ± 0.1^d^	1.3 ± 0.2^c^	0.5 ± 0.0^d^	0.6 ± 0.0^d^	0.4 ± 0.1^d^	<0.01	<0.01	<0.01
22:5n3 DPA	6.8 ± 0.1^a^	2.7 ± 0.5^c^	4.6 ± 0.7^b^	1.9 ± 0.2^c^	4.2 ± 0.1^b^	1.0 ± 0.1^cd^	3.0 ± 0.5^bc^	0.7 ± 0.0^d^	1.8 ± 0.1^cd^	0.6 ± 0.0^d^	<0.01	<0.01	<0.01
22:6n3 DHA	13.0 ± 0.3^b^	7.7 ± 1.0^c^	16.7 ± 2.7^ab^	8.4 ± 0.6^c^	18.9 ± 0.5^a^	8.1 ± 0.8^b^	19.0 ± 2.1^a^	8.0 ± 0.7^c^	20.5 ± 1.4^a^	12.4 ± 0.7^b^	<0.01	<0.01	<0.01
Total n3 PUFA	26.5 ± 0.3	13.3 ± 0.0	26.9 ± 3.6	12.3 ± 0.0	27.0 ± 0.7	10.0 ± 0.0	24.7 ± 3.7	10.4 ± 0.0	26.1 ± 1.7	13.6 ± 0.0	0.6	<0.01	0.15
Total PUFA	39.1 ± 0.4	20.2 ± 3.0	39.2 ± 5.6	19.5 ± 1.7	42.6 ± 1.2	15.6 ± 1.4	40.2 ± 5.2	16.0 ± 1.1	39.8 ± 2.7	22.7 ± 0.8	0.46	<0.01	0.14
Total n6 LCPUFA	3.8 ± 0.1^c^	1.9 ± 0.1^c^	6.8 ± 1.2^b^	2.9 ± 0.1^c^	8.3 ± 0.4^ab^	2.8 ± 0.3^c^	8.7 ± 1.3^ab^	3.3 ± 0.2^c^	10.2 ± 0.6^a^	5.1 ± 0.1^b^	<0.01	0	<0.01
Total n3 LCPUFA	24.8 ± 0.3	12.5 ± 1.8	24.2 ± 3.5	11.8 ± 1.0	25.8 ± 0.6	9.8 ± 0.9	23.9 ± 3.7	9.3 ± 0.8	23.3 ± 1.7	13.4 ± 0.8	0.67	0	0.06
n3:n6 PUFA ratio¶	2.1 ± 0.0^a^	1.9 ± 0.0^a^	1.8 ± 0.0^b^	1.7 ± 0.0^b^	1.7 ± 0.0^b^	1.7 ± 0.0^b^	1.5 ± 0.1^bc^	1.8 ± 0.0^b^	1.4 ± 0.0^c^	1.4 ± 0.1^c^	<0.01	0.07	<0.01
DHA:EPA ratio	3.3 ± 0.2^c^	4.2 ± 0.3^c^	7.6 ± 0.9 ^bc^	6.5 ± 0.4 ^bc^	9.9 ± 0.6 ^b^	11.6 ± 2.1^b^	14.6 ± 2.4^b^	16 ± 0.7^b^	34.2 ± 1.7 ^a^	31 ± 4.9 ^a^	<0.01	0.43	0.84

SFA, saturated fatty acids (sum of all fatty acids without double bonds); MUFA, monounsaturated fatty acids (sum of all fatty acids with a single bond); PUFA, polyunsaturated fatty acids (sum of all fatty acids with ≥2 double bonds); n6 PUFA, omega 6 polyunsaturated fatty acids (18:2, 18:3, 20:2, 20:3, 20:4, 22:4, 22:5); n6 LCPUFA, omega 6 long chain polyunsaturated fatty acids (20:2, 20:3, 20:4, 22:4, 22:5), n3 PUFA, omega 3 polyunsaturated fatty acids (18:3, 18:4, 20:3, 20:4, 20:5, 22:5, 22:6); n3 LCPUFA, omega 3 long chain polyunsaturated fatty acids (20:3, 20:4, 20:5, 22:5, 22:6); EPA, eicosapentaenoic acid; DHA, docosahexaenoic acid, and n3:n6 ratio calculated for total n3 PUFA: total n6 PUFA. Mean values across the row not sharing a common superscript were significantly different as determined by Tukey’s HSD test, P<0.05. ND, not detectable (<0.1% of total fatty acids).

§ in many cases ± 0.0 (S.E) values are rounding error.

¶We first computed the ratio for each replicate and then computed an average and SEM for the diet treatment.

‡ Significance probability associated with F-statistics.

With respect to MUFAs in the fillet, total MUFA content and all fractions, except for 16:ln9, had significant time effects and generally showed a higher content at the middle (42 days) of the experiment compared to the end of the experiment (84 days). The concentrations of 16:1n7, 18:1n7, 20:1n9, 20:1n7, and total MUFAs were significantly affected by dietary treatments and time. Fish fed the Sc0 diet displayed the highest amount of MUFA, which was directly related to the MUFA content in the experimental diets. Irrespective of the diet, oleic acid (18:1n9) was the most abundant monounsaturated fatty acid (MUFA) in the fillet.

Most of the individual polyunsaturated fatty acids (PUFAs) varied greatly among five dietary treatments and time. Regarding n6 fatty acids, all n6 fractions including total n6 PUFA content had significant time effects and were higher at the end of the experiment (84 days) than at the middle (42 days). The total n6 PUFA content was not affected by the diet (P = 0.43) but showed a significant time effect and interaction between diet and time (P<0.01). From 42 days to 84 days, total n6 PUFA content increased by 12.6% in fish fed the Sc0 diet, and by 16.1% in fish fed the SC100 diet. The Sc0 fed fish contained the highest amounts of 18:2n6, 18:3n6, and 20:3n6. In contrast, fish fed the Sc0 diet had significantly decreased amounts of 22:5n6 compared to fish fed the Sc25, Sc50, Sc75, and Sc100 diet. At the end of the experiment, concentrations of 20:4n6 in the fillet did not differ among five dietary treatments (P = 0.6).

With respect to the n3 fatty acids, most of the n3 PUFA including 22:6n3 DHA and 20:5n3 EPA were significantly influenced by the dietary treatment or time or both. The n3 PUFA content was generally higher at the end of the experiment (84 days) than at the middle (42 days). For example, from 42 days to 84 days, the 22:6n3 DHA content increased by 13.0% in fish fed the Sc0 diet, and by 20.5% in fish fed the SC100 diet. Tilapia fed the Sc100 diet had the highest contents of 22:6n3 DHA in the fillet lipids at the end of the experiment, and reflected the higher 22:6n3 DHA supplied by this diet. Furthermore, increasing the levels of Sc (Sc50, Sc75 and Sc100), which corresponded to reduced levels of fish oil, resulted in significant increases in the fillet 22:6n3 DHA compared to the control diet (Sc0) at the end of the feeding experiment. Tilapia fed the Sc0 diet had significantly increased amounts of 18:3n3 compared to the Sc75 and the Sc100 diet. They also exhibited significantly increased amounts of 20:5n3 EPA and 22:5n3 DPA compared to the four Sc inclusion diets due to a higher concentration of 18:3n3 in the Sc0 diet. However, the amounts of total n3 PUFA, total PUFA, and total n3 LC PUFA were not significantly different (P>0.01) in any of the diets. The n3:n6 and DHA:EPA ratio were significantly influenced by the dietary treatment. The n3:n6 PUFA ratio in the fillet was the highest in fish fed the Sc0 diet. Tilapia fed the Sc100 diet had significantly increased DHA level, hence increased DHA:EPA ratio compared to the Sc0, Sc50, and Sc75 diet.

**Fig 1:**
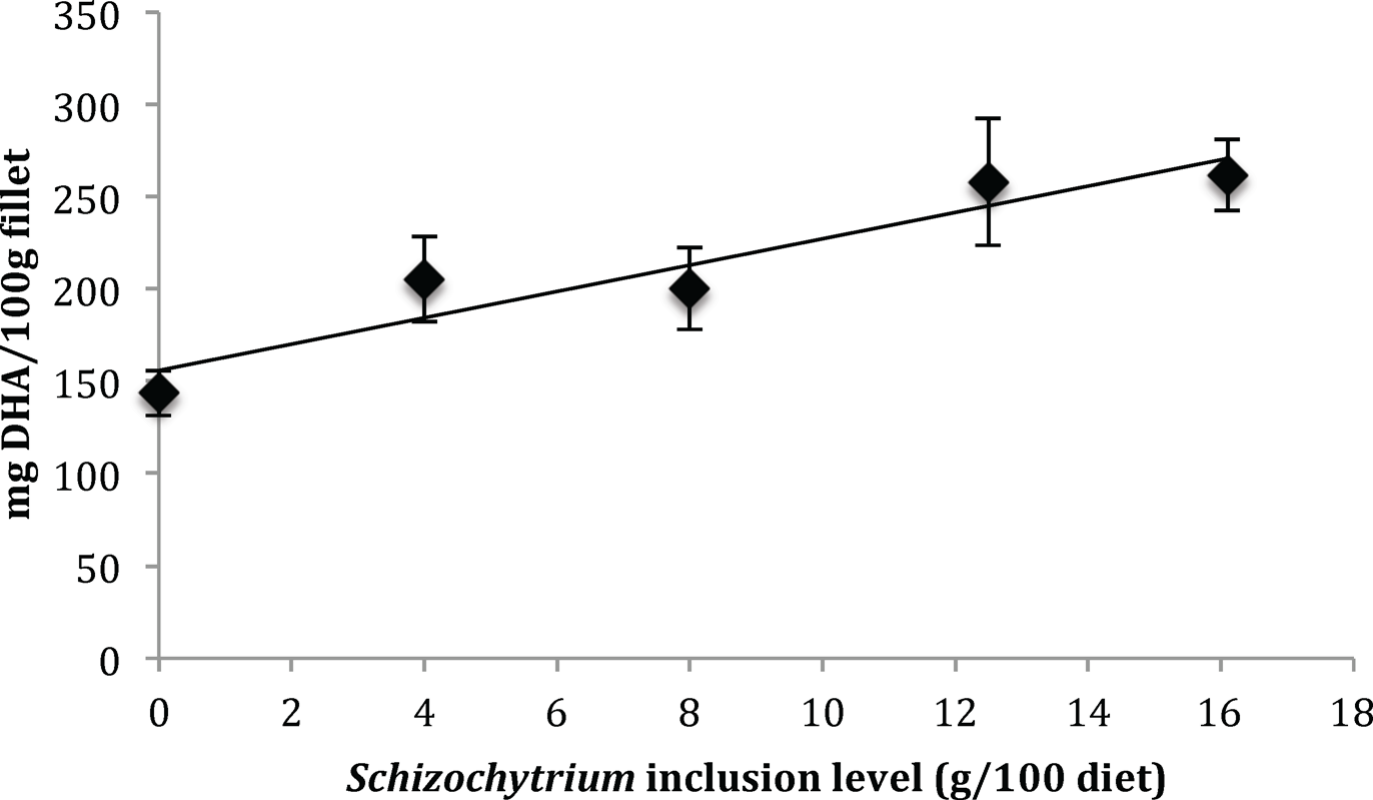
Linear correlation between the Sc inclusion levels in diet and DHA (mg/100g fillet) deposition in fish fillet. Each point represents the average value (±SE) of three tanks per diet. The amount of DHA is described by the function of y = 7.1423x + 155.8. The correlation coefficient (r) was 0.9459 (*p*<0.01).

Amounts of 22:6n3 DHA deposited in the fish fillet (mg/100g fillet) significantly increased in fish fed the Sc100 diet compared to the Sc0 diet (Table 7). With increasing Sc inclusion levels in the diet, the deposition of DHA in the fillet increased from 143.5 mg/100g for the inclusion level of 0 g Sc /kg diet (Sc0 diet) to 261.8 mg/100g for the inclusion level of 161 g Sc /kg diet (Sc100 diet). The relationship between Sc inclusion level in the diet and deposition of DHA (mg/100g) in the fillets of tilapia was positively correlated (y = 7.1423x + 155.8; r = 0.9459; P<0.01; Fig 1).

**Table 7:** Amounts of lipid and major n3 and n6 PUFA in the fillet (wet weight basis) of Nile tilapia fed experimental diets for 84 days; average ± SE for 15 fish per diet (5 fish/replicate with 3 replicates/diet).

			Fillet			F value	P value
Composition	Control (FO)	SC25	SC50	SC75	SC100		
Lipid (g/100g)	2.2 ± 0.20	2.2 ± 0.74	2.3 ± 0.26	2.3 ± 0.18	2.2 ± 0.06	0.03	0.99
18:2n6 LA (mg/100g)	96.7 ± 13.9	70.7 ± 14.1	77.5 ± 11.2	76.2 ± 11.2	55.1 ± 7.2	1.6	0.24
20:4n6 ARA (mg/100g)	3.8 ± 0.6	3.3 ± 0.4	3.1 ± 0.2	2.9 ± 0.3	2.7 ±0.1	1.2	0.36
18:3n3 ALA (mg/100g)	13.6 ± 2.4	6.4 ± 1.4	6.9 ± 1.2	6.4 ± 0.9	3.2 ± 0.4	6.8	0.006
20:5n3 EPA (mg/100g)	52.0 ± 7.9^a^	22.1 ± 5.0^b^	20.5 ± 3.4^b^	16.1 ± 2.9^b^	5.9 ± 0.5^c^	13.5	<0.01
22:6n3 DHA (mg/100g)	143.5 ± 12.2^c^	205.3 ± 23.0^b^	200.4 ± 22.3^b^	258.0 ± 34.3^a^	261.8 ± 19.3 ^a^	4.2	0.02

LA, linoleic Acid; ARA, arachidonic acid; ALA, alpha linolenic acid; EPA, eicosapentaenoic acid; DHA, docosahexaenoic acid. Mean values across the row not sharing a common superscript were significantly different as determined by Tukey’s HSD test, P<0.05.

## Discussion

This study is the first report of improved feed utilization indices, weight gain, and beneficial fatty acid profiles in Nile tilapia when fish oil is fully replaced in tilapia diets by 16% of dried whole-cells of a marine microalga species, *Schizochytrium* sp. (Sc). Additionally, SGR and survival rates were high and showed no significant difference among all diets at the experiment’s end. These results indicate that *Schizochytrium* sp. provides a high-quality substitute for fish oil and a supplement of long-chain polyunsaturated fatty acids (PUFA), especially 22:6n3 DHA, that improves n3LC PUFA deposition in Nile tilapia fillets. These results also point to the possibility of formulating ecologically and socially sustainable aquafeeds, with greatly reduced or no fish oil (from marine fisheries) and without having to switch to vegetable oils (from industrially farmed crops). Commercial realization of this potential will require advances in strategies to reduce non-renewable inputs (e.g., inorganic fertilizers and fossil fuels) and monetary costs of large-scale production of marine microalgae.

Regarding improved growth, fish fed the Sc100 diet exhibited significantly higher final weight and weight gain compared to those fed the Sc0 diet. The specific growth rate SGR (%/day), however, did not differ among all diets, which is likely due to slight differences (not significant) in the initial body weight among treatments, and subsequently the final body weight and thereby the calculated weight gain was higher in fish a higher initial body weight, and the SGR remained almost unaltered by the diets. During this 84-day experiment, mean SGR ranged from 3.3 to 3.5, fitting with literature values for the exponential growth phase [24,25].

Our results for improved weight gain may seem surprising in light of previous reports that a high dietary level of n3 LC PUFA depressed growth [26] or did not increase growth performance of tilapia [27]. We have identified two possible reasons for this difference between our results and previous research. Firstly, elevated levels of Sc exhibited improved FCR, consistent with our prior observation that the nutrient of Sc is highly digestible to tilapia [19]. Thus, the better feed conversion by Nile tilapia fed the Sc100 dietary nutrient was reflected in their improved weight gain; and this is similar to a recent report that dietary inclusion of Sc stimulated muscle or tissue development of Atlantic salmon [28]. Our observed effect of Sc on the growth of tilapia is also consistent with findings in shrimp and barramundi, which demonstrated an algal derived DHA stimulated growth performance [29,30]. Our followup studies are examining effects of Sc-inclusion diets in tilapia fed up to market size.

Secondly, dried whole cell Sc may contain high levels of a micronutrient [31,32], such as the caroteniod astanxanthin and bioactive compounds, which we did not measure but could have contributed to the growth benefit of whole cell Sc.

The relatively high values of FCR and low value of PER in fish fed the control diet, compared to fish fed Sc inclusion diets, could be due to lower nutrient digestibility of the control diet containing fish oil compared to Sc-inclusion diets. In our previous digestibility study with Nile tilapia [19], we confirmed that the Sc diet, with high DHA amounts (302g kg^-1^ of total fatty acids), did not depress the digestibility of protein, amino acids, and unsaturated fatty acids in tilapia compared to a reference diet containing only fish oil. We also found high digestibility of lipid and all unsaturated fatty acid fractions in the Sc diet compared to the reference diet containing fishmeal and fish oil, and suggested this may be because tilapia more easily digest the polar fraction of phospholipids prevalent in marine microalgae than they digest neutral class lipids (triglycerides) prevalent in fish oil (reviewed in [19]).

Depositions of total n3 PUFA and total n3 LC PUFA did not differ among all dietary treatments. Thus, even the diet replacing 100% of the fish oil with Sc (Sc100) did not reduce these totals compared to the control diet (Sc0). Total n3 LC PUFAs in tilapia fillets increased from 13% to 20.5% of total fatty acids when we fed the Sc100 diet that replaced all the fish oil with dried Sc (at 16% of the Sc100 diet) compared to the Sc0 diet containing only fish oil (at 9% of the Sc0 diet), although this difference was not significant.

Length of feeding time significantly influenced the deposition of most of the n3 PUFA, including 22:6n3 DHA and 20:5n3 EPA. The fillet contents for all the n3 and n6 PUFA content was higher at the end of the experiment (84 days) than at the middle (42 days), whereas fillet contents of SFA and MUFA fractions was lower at 84 days. These results fit with the understanding that the fatty acid composition of the fish is largely determined by the digestible fatty acid intake of the animal. In fatty fish, the overall change in the fatty acid profile is determined by triglycerides while in lean fish, like tilapia, the muscle lipid is dominated by phospholipids (75.5%) [33,34,35]. Our prior results for digestibility of whole cells of Sc in tilapia showed that 22:6n3 DHA phospholipid is highly digestible (93%) [19]. The high digestibility of 22:6n3 DHA is reflected in this study, given that tilapia fed Sc100 deposited the highest amount of 22:6n3 DHA in muscle at the end of the experiment (84 days).

Our results regarding fillet deposition of two important n3 LC PUFAs, DHA and EPA, can be explained by prior research and the relative abundance of these two fatty acids in Sc. Regardless of the treatment, 22:6n3 DHA was the major n3 LC PUFA found in the fillet lipids, supporting the conclusions of [21] that DHA, rather than EPA, was preferentially incorporated into the fillet of tilapia. This result along with the significantly higher weight gain of tilapia fed the Sc100 diet, compared to the Sc0 diet, leads us to propose the following hypothesis: the Sc100 diet, containing elevated levels of dietary 22:6n3 DHA, reduces energy expenditure that would have been spent on *de novo* DHA biosynthesis and consequently supports the growth performance of tilapia. Thus, future research is needed to elucidate the mechanism by which dietary 22:6n3 DHA affects energy expenditure, fatty acid metabolism, and growth in tilapia.

The Sc0 diet’s level of 20:5n3 was 11.1% of total fatty acids but tilapia fed the Sc0 diet deposited a lower level of 20:5n3 in the fillet (3.9% of total fatty acid), suggesting that 20:5n3 EPA will likely be extensively oxidized in Nile tilapia. This is in agreement with the notion that fish, including tilapia, selectively use 20:5n3 EPA as substrate for β-oxidation to produce sufficient energy [21].

Using dried whole-cells of a marine microalga as a rich source of LC n3 PUFA, instead of an extracted oil, offers several benefits regarding the human nutritional value of tilapia fillets. One benefit is reduced lipid oxidation in fillets, given that marine-derived fish oil is highly susceptible to oxidation. Aquafeeds enriched with fish oil increase the susceptibility of fish fillets to lipid oxidation, yielding undesirable lipid oxidation products that adversely affect fillet quality [36,37]. The benefit of using DHA-rich whole cell *Scizochytrium* sp. biomass in fish feeds is the natural encapsulation provided by the cell wall, which should protect valuable fatty acids from exposure to oxidative agents. Moreover, this alga is a potential source of natural antioxidants such as astaxanthin and carotenoid pigments [31,38], which could be readily deposited in fish tissue and enhance oxidative stability.

Inclusion of dried whole-cell Sc in aquafeeds offers a second human nutritional benefit of the fish product. Recent mammalian studies have revealed higher anti-inflammatory and immuno-enhancer properties of *Schizochytrium* sp., and attributed these findings to the docosapentaenoic acid (DPAn6) and DHA present in high levels in this microalga [38]. Such higher biological activity could also occur in tilapia fed diets with dried whole-cell Sc, and could translate to a human nutritional benefit of eating the fillet.

## Conclusion

This is the first study to use Sc as a complete fish oil replacement for Nile tilapia. Total replacement of the fish oil used in commercial diets with dried whole-cell Sc resulted in significantly higher weight gain (g) and protein efficiency ratio (PER), and lower (improved) feed conversion ratio (FCR) and feed intake when compared to the control feed containing fish oil (Sc0); and no significant change in specific growth rate and survival rate among all diets. Our results are interesting for tilapia farming by showing how microalgae inclusion can help optimize the composition of tilapia starter and juvenile feed. Diet formulation with dried whole-cell Sc is also an innovative strategy to raise the n3 LC PUFAs level and thus rebalance the n3:n6 and DHA:EPA ratios in farmed tilapia, which will benefit the health of human consumers. In our study, complete substitution of fish oil with Sc led to n3:n6 and DHA:EPA ratios of 1.4:1 and 34.2:1, respectively in tilapia fillets, suggesting the relative ease by which inclusion of whole cell Sc in tilapia diets can manipulate and tailor the n3 LC PUFA composition of tilapia fillets. However, in this study we used juvenile fish over a narrow growth range. Feed manufacturers can explore this approach to develop aquafeeds for aquaculturists aiming to cater to the consumer willing to pay a premium for health enhanced foods. Niche markets may therefore be developed for these nutritionally enhanced tilapia fillets.

In order for such nutritionally enhanced tilapia to succeed in the market, researchers have to find the ways to cut the high production cost of microalgae [39,40]. Towards this end, we are now focusing our research on reducing production costs by using organic waste streams, such as on-site fish culture effluent, as a partial replacement for expensive, external inputs of inorganic compounds normally used to grow microalgae. Furthermore, ongoing research to lower costs of producing and processing microalgae for biofuel and human nutraceuticals may lead to higher market availability and lower price of this microalga in the near future [41,42,43]. Our findings also open up a pathway to reduce or eliminate ingredients extracted from marine forage fish and from industrially farmed crops in aquafeeds. Our lab is now investigating combinations of different marine microalgae to reduce or eliminate fish oil, as well as fishmeal, in aquafeeds, towards the goal of improving life-cycle ecological and social sustainability of aquafeeds.
